# Strategies to facilitate safe sexual practices in adolescents through integrated health systems in selected districts of Zimbabwe: a mixed method study protocol

**DOI:** 10.1186/s12978-020-0862-y

**Published:** 2020-01-31

**Authors:** Wilfred Njabulo Nunu, Lufuno Makhado, Jabu Tsakani Mabunda, Rachel Tsakani Lebese

**Affiliations:** 1 Department of Public Health, School of Health Sciences, University of Venda, Thohoyandou, South Africa; 2 Department of Environmental Science and Health, Faculty of Applied Sciences, National University of Science and Technology, Bulawayo, Zimbabwe; 3 School of Health Sciences, University of Venda, Thohoyandou, South Africa

**Keywords:** Adolescents, Health system, Safe sexual practices, Strategies, Umguza, Mberengwa, Zimbabwe

## Abstract

**Background:**

Zimbabwe has the highest teenage pregnancy rate in Sub Saharan Africa. Human Immunodeficiency Virus (HIV) and Acquired Immunodeficiency Syndrome (AIDS) prevalence in adolescents that are from tribes that perform cultural initiations and subscribe to certain norms are higher than the national prevalence which is estimated at 12% (18 and 13.6% respectively) in Zimbabwe. Indigenous Health Systems (IHSs) and Modern Health Systems (MHSs) in Zimbabwe run parallel thereby introducing challenges in the management of adolescent sexual health due to conflicts. This study seeks to develop strategies that will facilitate the integration of IHSs and MHS in Mberengwa and Umguza districts.

**Methods:**

This research will be conducted in two phases. The first phase would utilise a concurrent triangulation mixed methods design with both qualitative and quantitative approaches. The findings from the qualitative and quantitative approaches would be merged through a comparison of findings side by side. The second phase would focus on the development and validation of strategies that would facilitate the integration of IHSs and MHSs. The Strength, Weakness, Opportunity and Threat (SWOT) analysis would be applied on interfaced findings from phase one. The Basic Logic and the Build, Overcome, Explore and Minimise (BOEM) models would then be used to develop strategies based on the SWOT findings. The developed strategies would be validated through the application of Delphi technique and administration of checklist to selected key stakeholders through organised workshops.

**Discussion:**

There have been no known studies found in the literature that explores the possibility and developed strategies of integrating IHSs and MHSs so as to promote safe sexual practices in adolescents. Most programs on sexual health have ignored the role of IHSs and MHSs in influencing safe sexual practices leading to them failing to attain desired goals. A lot of emphases has been targeted at minimising the spread of Sexually Transmitted Infections (STIs) through advocating for utilisation MHSs rather than focussing on an integrating systems that are meant to manage Adolescent Sexual Health (ASH) related issues. The study protocol was approved by the University of Venda Ethics Committee Registration (**SHS/19/PH/17/2608**) on the 26th of August 2019.

## Plain English summary

In Zimbabwe there is existence and recognition of two health systems that are the modernised health systems that is mannered by trained health service providers and the indigenous one that is manned by traditional healers which leverages on culture and customs. It has been noted that adolescents from communities that observe cultural practices and norms have a higher prevalence of Sexually Transmitted Infections and pregnancy. The two mentioned health systems work in parallel and do not complement each other. This study, therefore, seeks to develop strategies that would facilitate the integration of these two health systems so as to improve Adolescent Sexual Health outcomes. The study is going to be conducted in two phases that is, the first phase would involve collection and analysis of data from key stakeholders in adolescent sexual health. The second phase would leverage on the findings from phase one to develop strategies that would facilitate the integration of the two systems so that they complement each other. Furthermore, in this phase, the developed strategies would be validated through stakeholder engagement. This study, therefore, provides a window of opportunity for improvement of adolescent sexual health outcomes through complementary integrated systems.

## Background

Health Systems (HSs) play significant roles in shaping up peoples perspectives regarding different issues in life [1]. These are influenced by different characteristics of societies that presents with different contextual factors regarding Adolescent Sexual Health (ASH) [2, 3]. HSs arrangement are contextual and specific to a particular culture and society and this influences its performance regarding ASH issues [4, 5]. Human beings gather knowledge for two specific purposes in life, namely, meaning and survival [6]. Humans can, therefore, process and analyse knowledge and arrive at a decision whether or not to adopt or adapt certain strategies that would enable them to survive [1, 7]. It should be noted that adolescents are still at developmental stages, most of their decisions are influenced by the environment that surrounds them [8, 9]. They learn and shape their preferences using some experiences from their surrounding environment which in turn shapes their attitudes and behaviours towards their sexuality [8].

Some practices create a rifts between adolescents and their parents leading to no-dialogue on sexual issues as some practices predispose some populations to Sexually Transmitted Infections (STIs) [10, 11]. This breakdown in communication creates a challenge as adolescents are not given enough information relating to sexual health thereby predisposing them to risky sexual practices [10, 12]. A study conducted by Mavundla in 2009 on Xhosa men in South Africa, revealed that being circumcised was regarded as a rite of passage from childhood to manhood [13]. This is often confused by adolescents where they exhibit careless behaviour after circumcision as they deem themselves adults. This often leads to bad choices, risky sexual behaviours and often infection with STIs [13].

The National Adolescent fertility study (2015) reported that 58.4% of adolescent pregnancies in Zimbabwe were associated with cultural practices such as forced / early teenage marriage, traditional cleansing, wife pledging and Human Immunodeficiency Virus (HIV) cleansing [14]. Such practices (from Indigenous Health Systems (IHS)) are imposed on adolescents and have deleterious effects on their sexual health. In Mberengwa and Umguza Districts teenage pregnancy rate was at 17.7 and 23.6% respectively against the National average of 12% in 2012 [15]. These two districts still practice cultural initiations on adolescents and have high prevalence of STIs as compared to the national average [14, 16]. Adolescent HIV and AIDS prevalence in indigenous and cultural populations is higher than the national prevalence (18 and 13.6% respectively [17, 18].

In Zimbabwe there has been a joined up effort in trying to improve ASH [16]. There are programs such as condom distribution in schools, access to free family planning services and awareness campaigns conducted by health service providers [14, 19]. Though these services are available barriers such as religion, culture and adolescent unfriendly health institutions have reduced access to these services by adolescents [20]. In Zimbabwe it has been noted that adolescents in districts that rely mainly on IHS and practice cultural initiations have higher prevalence of STIs and teenage pregnancy [14]. Since Zimbabwean independence in 1980, the two health systems (IHS and MHS) have been recognised by the Government. However, collaborations remain extremely minimal with the two HSs running parallel [17, 18]. This has created tensions and conflicts as in most cases Traditional Health Practitioners (THPs) and Health care workers do not work together for the benefit of adolescents. In many instances where the traditions and beliefs of indigenous people are questioned they tend to shun away from MHSs. Majority of Zimbabweans rely on IHS for their health care.

There is scarcity of policies that have advocated for the integration and collaboration of IHS and MHS in Zimbabwe. Majority of Zimbabweans rely on IHS for their health care as it is affordable and accessible to many especially in resource constrained rural areas such as in Umguza and Mberengwa Districts [21]. Despite the fact that these systems are dealing with the same client (adolescents), there are minimal efforts that have been channelled towards ensuring that activities of these systems relating to ASH are integrated and delivered in an integrated manner [17, 18]. This could possible ensure effectiveness and efficiency of the two systems in improving ASH. There is therefore need to develop a strategy that would integrate the two health systems so as to improve ASH outcomes in the two selected districts with high teenage pregnancy and high prevalence of STIs. This study therefore seeks to develop strategies that will facilitate the integration of IHS and MHS in Mberengwa and Umguza districts in Zimbabwe so as to foster safe sexual practices in adolescents.

## Methods / design

### Research approach

**Fig. 1:**
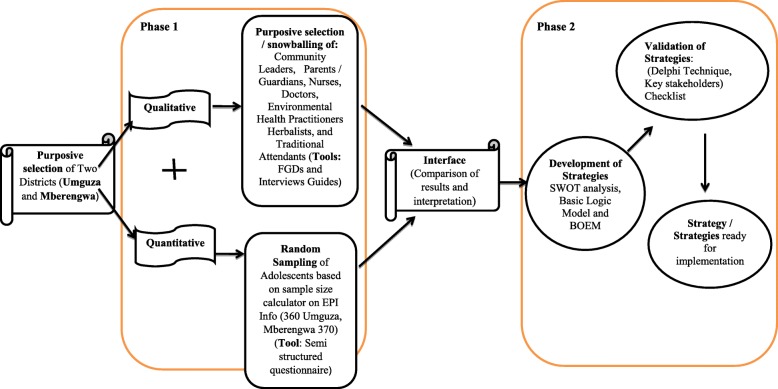
Research Approach

A research approach is a plan that guides the research process step by step. It outlines in a nutshell the critical steps that would be involved in the whole research and how they will be integrated together [22]. The research approach is informed by the research problem, objectives, methods and how the results would be integrated [22]. The study is going to be conducted in two phases. The first phase would gather the empirical data from responds. The data would be analysed and then inform the second phase that would involve the development and validation of strategies that would facilitate the integration of IHS and MHSs. The research approach is summarised in Fig. 1.

## Phase 1

### Phase 1 (a) systematic literature review

#### Review title

Health System Strategies and Adolescent Sexual Health. Systematic review using Rodgers Concept Analysis Framework. The summary of the review process is summarised on Table 1.

**Table 1:** Outline of Concurrent Triangulation Mixed Method Research Process

	Method	Objectives	Participants	Data Collection Method	Data Analysis
Phase 1 (Concurrent Triangulation Mixed Method)	*a) Systematic Literature review*	1. Review literature on the relationship between Health Systems Strategies and Adolescent Sexual Health issues guided by Rodgers evolutionary concept analysis framework. 2. Develop a Conceptual Framework that would guide a study that seeks to “Develop strategies to facilitate safe sexual practices in adolescents through Integrated Health Systems in Umguza and Mberengwa Districts in Zimbabwe”.	Quantitative and qualitative studies and reports that are published in English up to December 2018 in peer reviewed journals and are obtainable from Google Scholar, PUBMED, EBSCO and Science Direct**.**	Developed Data collection form guided by Rodgers Evolutionary Conceptual Analysis Framework	Thematic Analysis
	*b) Qualitative study*	3. Explore Indigenous Knowledge that influences sexual experiences; 4. Assess the role played by different stakeholders in communities that influence adolescent development and sexual experiences	Community Leaders / herbalists / Traditional Attendants, Health Service Providers (Doctors, Nurses and Environmental Health Practitioners involved in adolescent sexual health issues), Adolescents parents / legal guardians,	Interviews Focus Group Discussions	Thematic Analysis in MAXQDA
	*c) Quantitative study*	5. Establish the extent of influence of IHSs and MHS on adolescent sexual behaviours	Adolescents	Questionnaire with both open ended and closed questions	Cross Tabulations and Multiple Logistic Regressions in STATA Version 13 SE

##### Background of the review

Health Systems Strategies (HSSs) play a major role in ensuring access to Sexual Health (SH) services by adolescents and in turn impact on their SH outcomes [23]. Health Systems (HSs) have been defined as organisation of people, institutions and resources so as to ensure delivery of SH services to adolescents [24–26]. It is of importance to note that this age group is not totally independent in making their decisions therefore would rely on their environments to inform their decisions [26]. There are quite a number of strategies that have been implemented world-wide so as to improve Adolescent Sexual Health (ASH) outcomes [27–29]. Strategies in this study will be defined as a plan that is implemented within a HS so as to impact on SH outcomes of adolescents [30]. Despite implementation of quite a number of strategies adolescents still remain highly vulnerable, with high prevalence of Sexually Transmitted Infections (STIs), high incidence of teenage pregnancy resulting in high numbers of adolescents dropping out of schools [2, 26]. Dropping out of formal schools subjects adolescents to poverty particularly in Low and Middle Income Countries (LMCIs) where employment opportunities are hard to come by and there is stiff competition for the few available opportunities [31]. Concepts are not well understood when it comes to implementation of ASH programs leading to low demand and misinterpretation of such programs that are meant to improve their SH outcomes [23, 29].

##### Review question

What is the relationship between Health Systems Strategies and Adolescent Sexual Health is as presented in literature over time?

##### Specific objectives

- Review literature on the relationship between Health Systems Strategies and Adolescent Sexual Health issues guided by Rodgers evolutionary concept analysis framework.
- Develop a Conceptual Framework that would guide a study that seeks to “*Develop strategies to facilitate safe sexual practices in adolescents through Integrated Health Systems in selected Districts in Zimbabwe*”.

##### Methodology

***Inclusion criteria***

In this systematic review studies that present Health System Strategies that targeted Adolescent Sexual Health will be considered. This review would target studies and reports that are published in English up to December 2018 in peer reviewed journals the world over. The review would target original quantitative and qualitative research and reports obtained from Google Scholar, PUBMED, EBSCO and Science Direct.

***Exclusion criteria***

This systematic review will exclude all studies that focused on the relationship between Health System Strategies and their impact on other age groups (other than adolescents) were excluded from this study.

***Search Strategy***

The keywords: Adolescents, Health Systems, Sexual Health and Strategies will be used to search for relevant literature from Google Scholar, PUBMED, EBSCO and Science Direct.

***Methods of Review***

Titles and abstracts would be reviewed independently by at least two reviewers to identify articles and reports that would be relevant this systematic review. Disagreements would be resolved through dialogue between the reviewers. Full texts of these articles and reports that meets the inclusion criteria would also be reviewed by at least two reviewers with differences being ironed out through dialogue.

***Data Extraction and Synthesis***

A data collection form would be developed guided by Rodgers Evolutionary Conceptual Analysis Framework to facilitate uniform data collection by all reviewers on attributes, antecedents and consequences of Health System Strategies on Adolescent Sexual Health from the articles and reports that met the inclusion criteria. Collected data would then be compared and any deviations addressed through dialogue between the reviewers so as to reach a consensus. Findings from the articles and reports would be coded and thematically analysed to identify and explain antecedents, attributes and consequences of HSSs on ASH.

***Quality Assessment***

A quality evaluation tool will be used to assess the quality of the selected studies in line with Rodgers’ Evolutionary Concept Analysis Framework [32]. Articles and reports would be assessed for clarity in the presentation of attributes, antecedents and consequences of HSSs on ASH [33]. Furthermore the AMSTAR tool for assessing methodological quality of systematic reviews will be used to assess the quality of the methods used in this systematic review [34].

#### Phase 1 (b) and 1(c) concurrent mixed method

##### Study design

A concurrent triangulation mixed method design would then be conducted. This design would utilise both qualitative and quantitative approaches to collect and analyse data from respondents. This study design would seek to gather complementary and yet different data on the topic which would be obtained from different participants which would then inform the development and validation process of the strategies [35]. There is need to gather data in such a way that the findings are comprehensive as these are the basis for development of strategies. This design is appropriate as the qualitative and quantitative data would complement each other in explaining different data papers in relation with ASH. The results from the two methods (quantitative and qualitative) would then be interfaced, compared and interpreted. This would help in ensuring sound developed strategies are contextualised to solving problems that would have been explored in terms of the magnitude of influence. The objectives for this phase are summarised in Table 1.

##### Setting

The research would be conducted in two Districts, Mberengwa and Umguza. These districts were purposively selected as they are part of the few known districts that are highly cultural and have defined initiation schools for adolescents and most people still rely on IHS. The districts also have a higher prevalence of STIs and high teenage pregnancy. Other districts are modernised and the health care seeking behaviour of members in those districts have shown that they favour MHS as compared to IHS [4, 36].

**Mberengwa District**

**Fig. 2:**
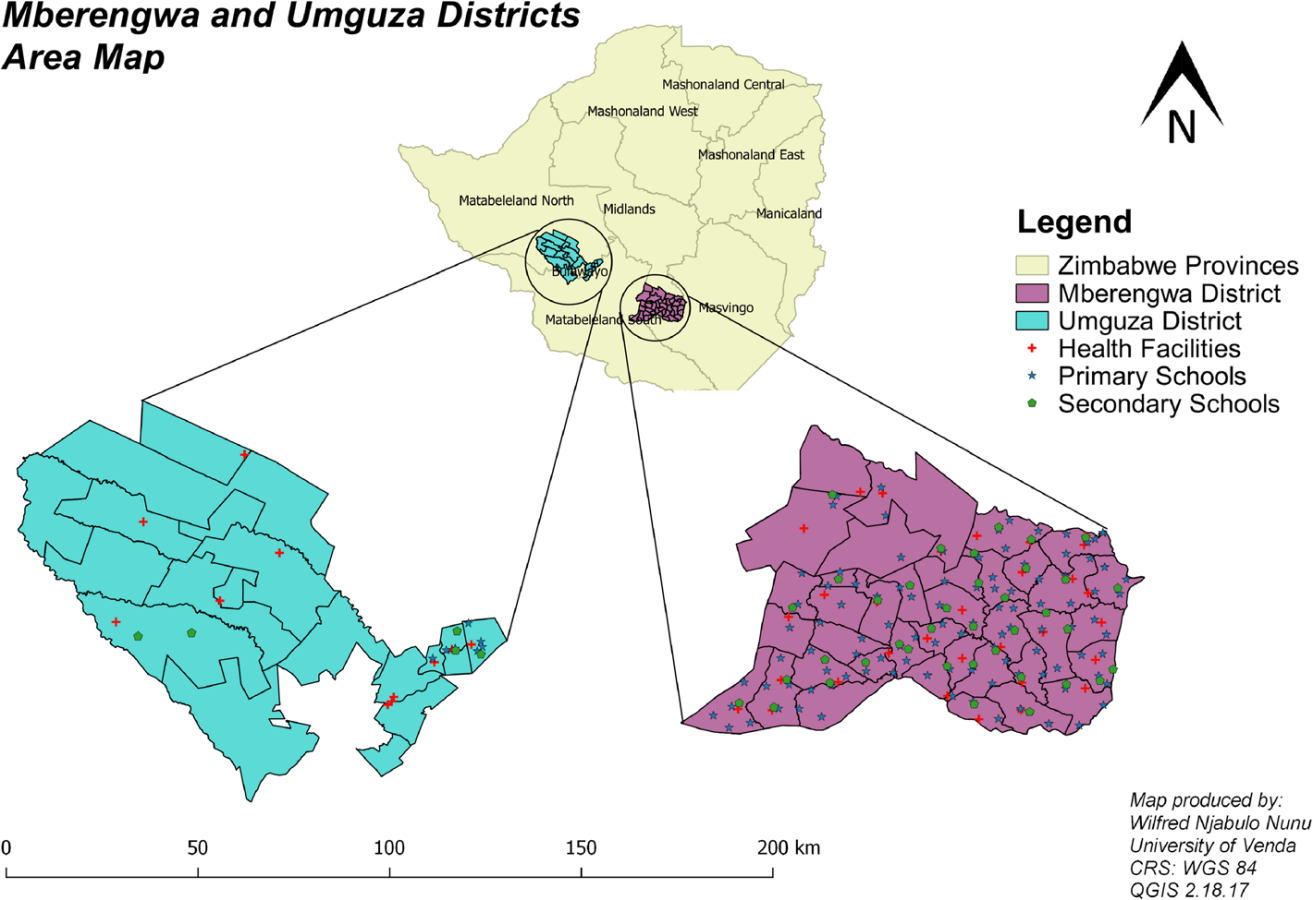
Mberengwa and Umguza Districts, Zimbabwe

Mberengwa district lies in the Midlands province in Zimbabwe. It is bounded by Gwanda to the west, Zvishavane to the north, Neshuro to the south and Chikombedzi to the east. This district is found in the Midlands province of Zimbabwe and has 37 wards, 32 health facilities, 104 primary schools and 38 secondary schools (see Fig. 2) [21]. Of these 32 health facilities, three are first level referral centres. These comprise of district and rural hospitals that deal with cases that cannot be taken care of at PHC level. Majority of people in this district rely on IHS. These systems run parallel to the modernised National Health Systems (NHS). The district is dominated by the Varemba tribe that are highly cultural [21]. Languages spoken in this district are Karanga and Ndebele and these languages are dialects of Shona and Ndebele Languages respectively. The total population was estimated at 200581 in the 2012 census. Of this population, 67,195 were aged between 5 and 14 years, 100,892 aged between 15 and 24 years. This means that adolescents could be comprising 50% and above of the total population. In this district there are several cultural activities that takes place including traditional initiation of adolescents which takes place in September in designated initiation schools. These initiation schools are run by traditional leaders and attendants who are deemed experienced. The district has STI prevalence of 19% in the age groups of 10–24 [14] where adolescents fall and the general HIV prevalence in the whole population is pegged at 16% [37]. According to the National adolescent fertility Study, Midlands Province has a teenage pregnancy prevalence rate of 17.7%. This rate is too high as some countries in developed countries have rates as low as 8 per 1000 (which could translate to around 2% or less of their teenage populations) [38].

**Umguza District**

Umguza district lies in Matabeleland North province and is made up of 18 wards, has 11 health facilities, seven primary schools and five secondary schools (as shown in Fig. 2). All the seven health facilities are PHC facilities. The district had an estimated population of 80,971 during the 2012 census. Of this population 21,133 people were aged between 5 and 14 years, 47,206 being aged between 15 and 24. Tribes found in this district are Xhosa and Ndebele both of which are classified as dialects of Ndebele. This district is highly traditional with an HIV prevalence of 18.9% in the general population as compared to the national average of 12% [39]. Teenage pregnancy in Matabeleland North Province (the province in which the district is housed) stands at 23.6% against the targeted 12% in 2020 [39]. The district is highly reliant on traditional health system as the district is largely rural with high unemployment levels [14]. Initiation schools for adolescents are held during winter that is, June and July every year. In these schools, activities such as male circumcision and labia elongation are conducted on male and female adolescents respectively.

#### Phase 1 (b) qualitative approach

A qualitative study would be conducted on key informants (Community Leaders / Herbalists, Traditional attendants, Health Service providers, and adolescent parents / guardians). Data would be collected from targeted participants in order to build themes on IHS and how it influences adolescent sexual experiences and how IHSs relates to MHSs. The qualitative study design would enable understanding of IHSs and how these impact on adolescent sexual experiences. The design would also aide the identification of potential strategies that could facilitate integration of IHSs and MHSs so as to improve adolescent sexual health outcomes.

##### Study population

These will involve community leaders, herbalists and traditional attendants, health service providers (Nurses, Doctors and Environmental Health Practitioners dealing with adolescent sexual health issues) and parents and legal guardians of adolescents.

##### Sampling

Two districts were purposively selected as these had high cases of teenage pregnancy and STIs as explained in section 2.2.2 above. The sampling process for the targeted populations is summarised in Table 2.

**Table 2:** Summarised sampling process for the qualitative approach

Study Population	Sampling Procedure	Sample Size	Instrument and Data collection Procedure	Data Analysis Plan
1. Community Leaders (16 Chiefs in Mberengwa and 5 chiefs in Umguza)	Purposive	21	Unstructured Interviews, recording of interviews using a tape recorder	Coding and Thematic analysis on MAXQDA version 14
2. Herbalists and traditional attendants	Snowballing	Unknown, therefore sample size would be determined by data saturation.	Unstructured Interviews, recording of interviews using a tape recorder	Coding and Thematic analysis on MAXQDA version 14
3. Parents and guardians	Purposive	5 Focus groups with at 10 participants. The groups would comprise of 5 females and 5 male participants.	Focus Group Discussion Guide, recording the conversations using a tape recorder	Coding and Thematic analysis on MAXQDA version 14
4. Health Service Providers	Purposive	Mberengwa has 32 Health facilities therefore one doctor (if available), one nurse and one environmental Health Practitioner involved in adolescent sexual health issues would be selected at least one in each category per facility, the sample size would depend on data saturation. In Umguza there are 5 health facilities Sample of 5 doctors (if available), 5 nurses and 5 EHPs	Unstructured Interviews, recording of interviews using a tape recorder	Coding and Thematic analysis on MAXQDA version 14

**Sample Size Sampling of Respondents**

The two districts have a total of 21 chiefs (16 in Mberengwa and 5 in Umguza). All these community leaders would be targeted for interviews. The number of herbalists and traditional attendants that would be participating in the study would be determined by data saturation. Most herbalists and traditional attendants are not registered with the government, therefore snowballing would ensure that there are not missed out. At least three health service providers in Umguza and Mberengwa health facilities would be recruited to participate in the study. Health service providers’ (Doctors, Nurses and Environmental Health Practitioners) representatives would be purposively selected that is only those that deal with adolescent sexual health issues in their respective health facilities. The researcher would strive to make sure that doctors (if available as some health facilities do not have doctors), Nurses and Environmental Health practitioners (these are often involved in information dissemination and conducting awareness campaigns on public health issues including adolescent sexual related) are represented in each facility through purposive sampling. Lastly parents and guardians of adolescents would be chosen purposively and would comprise those that would have adolescents in the age group of 10–19 years of age. These would participate in FGDs which would have 5 groups of 10 participants each. These groups would be heterogeneous comprising of 5 male and 5 female participants.

**Inclusion Criteria**

All community leaders and herbalists would be eligible to be part of the study. Parents with selected adolescents would also have an opportunity to participate in the study. Health care givers in health facilities that are involved in adolescent sexual health in any way would also be eligible to be part of the study.

##### Instruments

The FGD guide would be used to collect data from parents/ guardian of adolescents. Unstructured Interviews guides would be used for collecting data from community Leaders, Traditional Attendants, Herbalists and Health Service providers. These guides would be administered in either English, Ndebele or Shona (in line with the preference of the respondent) which are the main three main languages that are used in Zimbabwe. These guides would are semi structured with a series of questions that would guide the enquiry in line with objectives. Probes will be asked to seek clarification on issues that would be arising during the interviews.

##### Pre-test

A pre-test of the tools will be conducted in one ward in Mangwe district which is also rural so as to avoid contamination of sampling pools [40]. Three participants from health facilities and three community or traditional attendants would be interviewed in Mangwe District. Furthermore, one FGDs would be conducted with parents and legal guardians of adolescents in the same district. The responses would be transcribed, and analysed, and necessary adjustments made to the interview and FGD guides.

##### Data collection

In-depth unstructured interviews would be conducted on key informants (that is community leaders, herbalists and traditional attendants and health service providers). These interviews would be conducted in private places during the times that are convenient for both the interviewer and interviewees. These interviews would seek to understand the roles that community leaders and traditional healers play on adolescent sexual issues. The interviews would also probe and identify IKS in the community and how it influences adolescent sexual practices. The interviews would also probe and identify possible ways of integrating IHS and MHS. FGDs would be held with legal guardians and parents of adolescents. Prior appointments would be made to ensure targeted participants participate in FGDs. This would be done to try and build up from the interviews with key informants so as to gain more understanding and triangulate some of the identified IHSs. This would be done to gain more insight and possible complement and generate more information that could have been missed in the process of interviewing key informants. The unstructured interviews would be conducted either in English, Ndebele or Shona languages using a language editor to translate the questions depending on what the respondents propose. The type of language chosen from these three will depend on what the respondents themselves feel comfortable in using. These three languages are the key languages in Zimbabwe and the two districts use at least one of these languages (either the dialects of Ndebele or Shona and English). The interviews would be recorded using a digital tape recorder.

##### Qualitative data analysis

Data collected from key informant interviews and FGDs will be transcribed and thematically coded and analysed on MAXQDA using Braun & Clarke six step as presented by Maguire & Delahunt in 2017 [41]. These steps involve: becoming familiar with the data, generating initial codes, searching for themes, reviewing themes, defining themes and writing up. Transcription would be done verbatim and. Codes would be developed with the help of an independent coder [41]. Emerging themes would be identified and presented as findings and these would be interfaced with findings from the quantitative study design that would be running concurrently.

##### Trustworthiness

These are very important in qualitative research to ensure the robustness of the data collected and how it is interpreted. Observation of these characteristics in data collected ensures that objectivity is achieved and all researcher underlying values that could impact on the results would be reported so that they are considered when one is reading the research. Below are the considerations that will be made.

**Credibility**

Credibility is concerned with the extent to which the research methods prompt confidence in the truth of the data and its interpretation [42]. This ensures that the results of the research are believable. This is going to be ensured through usage of different techniques in data collection (triangulation) that is, FGDs, interviews administered to different categories of participants such as health service providers, traditional leaders/ healers, traditional attendants, herbalist and parents / guardians. These would ensure richness of information that would be gathered. These would be (FGDs and unstructured interviews) employed to gauge accuracy of findings with attention given to verbatim quotes and outliers.

**Dependability**

Dependability refers to a process of evaluating the quality of integrated processes of data collection, analysis and theory generation [35, 43]. To ensure that the study is robust in terms of recruitment of participants, collection and analysis of the data, the proposal would be subject to different academic boards that is Departmental Committee and School of Health Higher Degrees Committee for review of the merits of the proposal in ensuring it meets the minimum standards required. Data collection would also be done over a period of three months from January to March 2019 to ensure stability of data over time and conditions. Collection of data at different intervals will minimise information bias while assessing if participants will give similar information to the same questions at different periods of time.

**Conformability**

Conformability refers to the degree to which the results could be confirmed by others [43]. The researcher is going to maintain high level of objectivity in collecting and interpreting these results of the qualitative enquiry. The researcher is also going to reflect on his interests that could potentially influence judgement and these taken into consideration during data collection and analysis. Manuscripts of this study (including the protocol paper) are going to be subjected to external peer reviews through submission of manuscripts to peer reviewed journals. This will enable reviewers to scrutinise the methods and data analysis techniques used to interrogate the data. This would question whether findings are supported by the data collected and the data collection tools thereof.

**Transferability**

Transferability is defined as the extent to which findings of a study can be applied to other situations [35, 43].This often demonstrates the possibility of the findings of the study being applied to a wider population or to a different setting. Authors have argued that even though a case is unique it is applicable to a broader scope or a wider community [43, 44]. Sufficient detail about methods, data collection and analysis tools would be provided to ensure readers are well informed. This would enable them to assess the research objectively and be able to transfer it to different settings.

#### Quantitative approach

A quantitative survey that establishes associations between IHSs, MHS and subsequent adolescent sexual experiences would be conducted on recruited adolescents.

##### Study population

The target population under this method would be adolescents aged 10 to 19 years in Umguza and Mberengwa districts in Zimbabwe.

##### Sampling

**Sample size**

The population of adolescents was estimated at 68339 for Umguza and 168,087 for Mberengwa respectively. Adjusting the population using the growth rate of 1.56% per annum as reported by the Zimbabwe demographic profile gives an estimation of 73,840 adolescents for Umguza and 181,618 for Mberengwa respectively by the end of year in 2017. The sample size was calculated using EPI INFO at a margin of error of 5%, confidence level of 95% and a response distribution of 50% gave sample sizes of 360 in Umguza and 370 in Mberengwa respectively. This sample size calculator is a prepared calculator which takes into consideration level of confidence, margin of error and response distribution. It involves the researcher inputting the population and adjusting those three variables mention above to get a sample size in line with the research setting. This would then give a sample size.

**Sampling of participants**

Participants would be recruited using stratified systematic random sampling such that all the wards in the two districts are represented to ensure proportionate representation of males and females in the sample and to also ensure that all age groups participate in the of the study. Mberengwa has a total of 37 wards therefore 10 participants would be drawn from each ward whilst in Umguza (18 wards), 20 respondents would be drawn from each ward. The study would ensure a 50:50 representation of males and females in all samples. Participants would be identified from community registers that are kept by Headman in their areas of jurisdictions (at ward level). The registers collect have demographic characteristics such as date of births and sex of all members of specific families. This would enable the researcher to fish out adolescents from that register who are in the 10 to 19 year age group. These registers would be considered as sampling frames to guide the sampling process.

**Inclusion criteria**

All adolescents aged between 10 years and 19 years during the time data is collected would be eligible to take part in the study.

##### Instrument

A semi structured questionnaire would be used to collect data from the respondents. This tool was adapted from John Cleland’s Questionnaire for interview surveys on sexual and reproductive health of young people available in the WHO website [45]. The questionnaire for adolescents was structured and comprises of six sections. The sections would explore the following; Demographics; Practices; Reasons for engaging in sexual activities and extent of supervision; Role of identified IKSs and their relationship with sexual experiences; Role of health systems in shaping up their sexual experiences; Views of potential integration of IHS and MHSs. A language translator would be engaged to translate the tool into Ndebele and Shona versions which are the major languages in the country for easy understanding as all languages in the country are either dialects of Shona or Ndebele. The questionnaire would have both open ended and closed questions.

##### Pre-test

Pretesting of the questionnaire would be done on 10 randomly selected adolescents in Mangwe District. After the pre-test, participants would be asked about the whole process of data collection and the nature of questions asked. Their input would lead to fine tuning of the instrument if need be. These respondents would not be included in the main study.

##### Validity

Validity is defined as the extent to which a concept accurately measured in a quantitative studies [42]. There is need to consider content validity which ensures that the designed data collection tools adequately cover all the content that it should with respect to the variables that are being probed. Content validity of the semi structured questionnaire will be ascertained through pretesting, standardisation and refinement. Face validity is also critical, where experts are asked their opinion about whether the proposed semi structured questionnaire measures the concepts that it is intended to. This proposal would go through various committees with experts and their feedback would be taken into consideration in fine tuning the data collection tool.

##### Reliability

Reliability is the consistency of the measurement, or the degree to which an instrument measures the same way each time it is used under the same condition with the same participants [42]. The data collection tool will be pretested prior to data collection and consistency checked using tests retest method to see whether there are deviations from data collected from same participants in a number of times. Data is going to be collected in a way that enhances precision and consistency, which will improve reliability. Data will be captured and cleaned in excel and checked for consistency and completeness before being imported to relevant software for analysis. Data collectors would also be trained so as to minimise inconsistencies in capturing of data.

##### Data collection

A researcher administered questionnaire with both open and closed questions would be used to collect data from adolescents. The questionnaire would be administered by the researcher with the assistance of two trained data collectors so as to increase response rate and also explain questions that might not be understood by the adolescents so as to capture as much accurate information as possible. The questionnaire would capture data on participants’ demographics, knowledge of adolescents towards STIs, sexual practices and test the role of identified IHS and MHS in influencing adolescents’ sexual experiences. Sampled adolescents would be followed up and the questionnaire administered. It should be noted that data collection would be done during term (October 2019 to January 2020) when most adolescents would be going to school. They will be followed up to their respective schools and these are run by the Ministry of education. Prior arrangements would be made with the School Heads regarding venues for data collection and each questionnaire would take approximately 10–15 min to administer.

##### Quantitative data analysis

Multiple logistic regressions and Cross tabulations between identified IKS with identified sexual experiences or practices would be done to establish the extent of influence of IHS and MHS on ASH. Demographic information such as age, economic status, religion, culture will be presented quantitatively and multiple logistic regressions performed to assess the relationship of these variables with adolescent sexual experiences. The analysis would done using STATA Version 13 SE.

#### Interface

Findings from the Qualitative and Quantitative approaches would be merged and compared side by side. This would enable for triangulation and comprehensive analysis and capturing of findings in a way that reflects an overview of state of matters by comparing findings from different stakeholders that would have taken part in the study.

### Phase 2

This phase will involve development and validation of strategies that would facilitate the integration of IHS and MHS basing on findings from Phase 1. Development of strategies would involve the application of SWOT analysis, Basic Logic Model and BOEM. Validation would include application of Delphi Technique and the use of an adapted checklist that would be administered to key stakeholders to get their opinion on developed strategies. The development and validation approaches are summarised in Table 3.

**Table 3:** Development and Validation of Strategies

	Method	Objectives	Participants	Data Collection Method	Data Analysis
Phase 2	*a) Development of strategies*	1. Develop strategies that leverage on empirical evidence to enhance HSs performance regarding management of adolescent sexual issues	Analysis of Data from participants in phase 1	SWOT Matrix Basic Logic Model BOEM	SWOT analysis to determine the possible areas to facilitate the integration
	*b) Validation of developed strategies*	2. Validate the developed strategies	Experts Key Stakeholders	Delphi Technique Checklist	Expert feedback Quantitative and qualitative analysis of data from the checklist

#### Strategy development

##### SWOT analysis

SWOT analysis has been defined as a structured process that aims to identify and analyse strengths, weaknesses, opportunities and threats of different strategies and different plans that aim to achieve set objectives [46]. It is a conceptual framework that is aimed at identifying and appraising strengths, weaknesses, opportunities and threats of phenomena of interest [47]. The SWOT analysis conceptual framework would be applied to the findings from the triangulation mixed method study conducted in phase 1. This would enable the identification and analysis of internal factors (human resources, competence, financial costs and services) and external factors (political, economic, socio-cultural, technological, legal and environmental) that would be helpful or harmful in the facilitation of the integration of IHS into MHS would be identified so as to be overcome or manipulated. This would form the basis of strategy idea brainstorming and appraisal using the Basic Logic Model.

##### Basic logic model

The Basic Logic Model follows a sequence of critical stages that are deemed necessary when developing a plan or strategy [48]. There is need to leverage on the findings and outcomes of the SWOT analysis to brainstorm on possible strategies that could facilitate integration of IHS and MHS. These would then be appraised to estimate the resources needed for their implementation so as to ascertain feasibility. There would also be need to ensure identification of activities that have to be done to facilitate implementation of the proposed strategies and this would enable determination of whether or not there is sufficient resources to implement the proposal. The strategy proposals would also be appraised based on the expected outputs, the short and long term outcomes as well as the perceived impacts. After this stage only ideas or strategy proposals that would be deemed feasible would then be subjected to the next stage the build, overcome, explore and minimise (BOEM) model in the next stage.

##### BOEM model

**Fig. 3:**
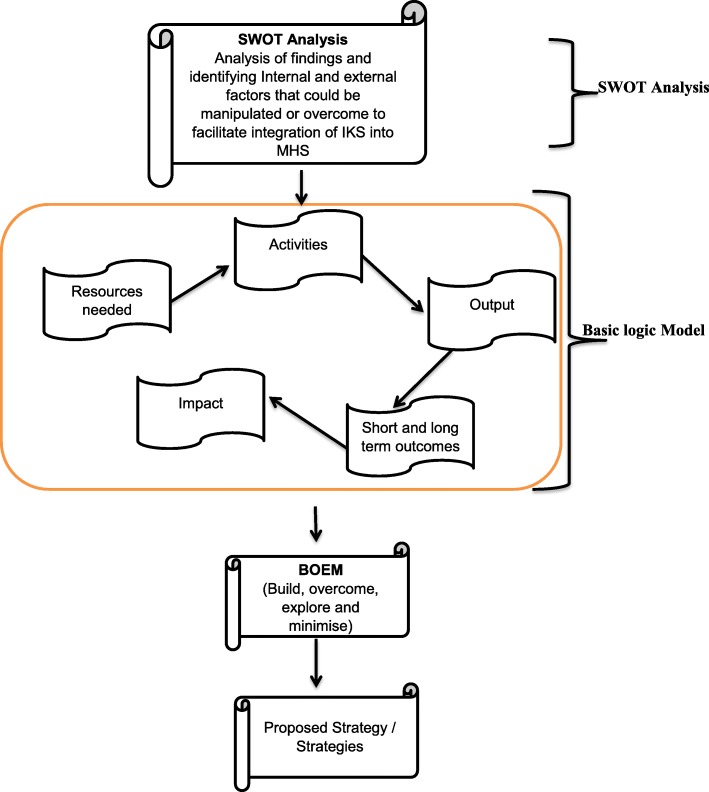
Strategy development flow chart

After subjecting the ideas that have to be developed to the basic Logic model, the BOEM model would then be applied. This model leverages on building a strategy that overcomes threats and weaknesses of the current systems at the same time exploring opportunities that would best support the achievement of objectives. Strategies would be built in such a way that minimises chances of them failing to attain the desired goals. The strategy development process is summarised in Fig. 3.

#### Validation of strategies

Validation of strategies aims at determining feasibility, applicability, acceptability and sustainability of these strategies in attaining desired goals [48]. It is critical that key stakeholders evaluate the developed strategies to ensure that they do not undermine or violate values of different health system users. Strategy evaluation would follow two key stages. The first stage would involve the Delphi technique, whilst the second stage would involve administration of a checklist to specific key stakeholders.

##### Delphi technique

The Delphi technique is a systematic, interactive method that is used in forecasting into the future regarding proposed methods, strategies and their likely impact they could have if they are implemented [49]. The objective of the method is to seek expert opinion on the developed strategies or implementation plans and forecast their likely impact in attaining set goals and objectives and their appropriateness [50]. This method is suitable in guiding strategy development in this research. Between 5 to 20 experts specialising in IHS, HSP and adolescent sexual health will be recruited. The experts would have to have extensive knowledge on the subject of interest as proven by their academic and scholarly background and they will be purposively selected. These experts would be briefed of the findings from the Triangulation mixed method study, the SWOT analysis, the Basic Logic Model and the BOEM model and the subsequent developed strategies. They will then be tasked to critique the developed strategies basing on the context and whether they have the ability to facilitate the integration of IHS and MHS for better adolescent sexual health outcomes. The experts feedback would be used to fine tune the strategies in preparation for validation by key stakeholders.

##### Key stakeholder consultation

**Fig. 4:**
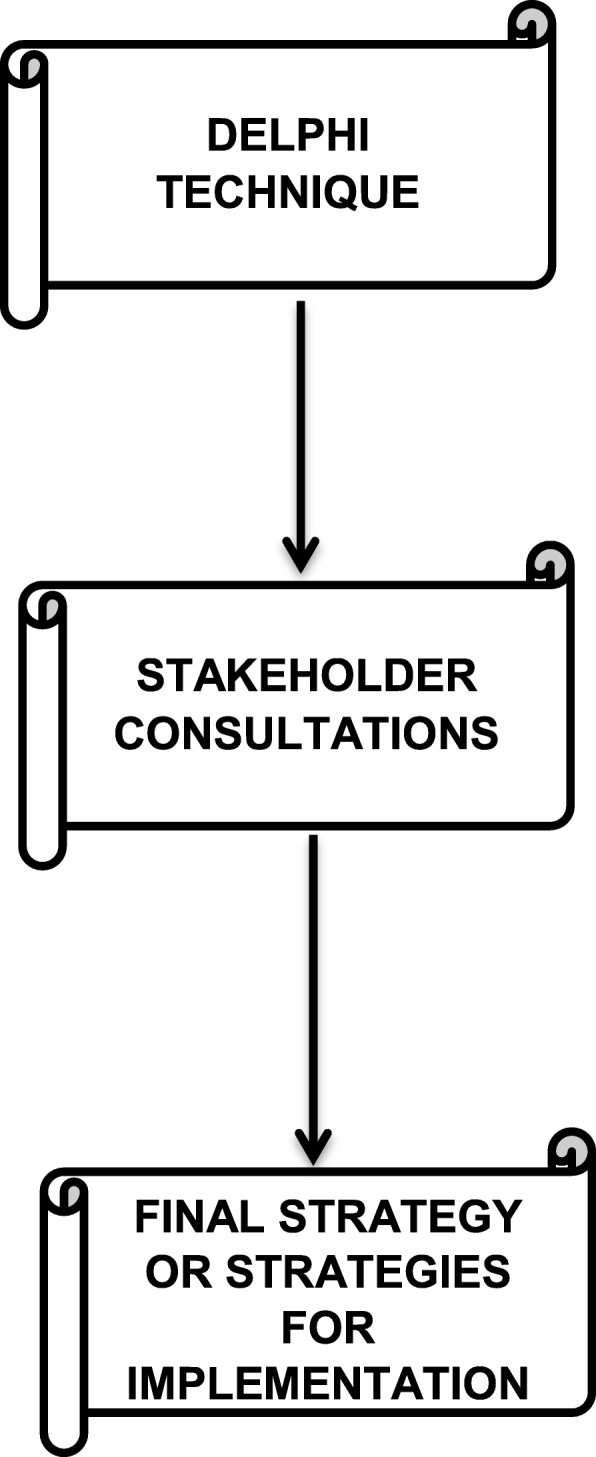
Strategy validation process flow chart

These will include Ministry of Health and Child Care representatives, Ministry of Education representatives, health service providers, community leaders, herbalists, adolescents and their parents. A total of 100 stakeholder representatives was calculated using a sample size calculator on EPI INFO. This sample size was calculated using a 95% level of confidence with a confidence with of 10% and an expected value of attribute of 50%. This gave a sample size of 96 which was then rounded off to 100. The researcher would ensure that 50 of the participants would be from Umguza district and the other 50 from Mberengwa. All key stakeholders would be recruited using stratified random selection to ensure that the targeted categories of key stakeholders are all represented. A checklist having 14 questions would be used to gather data on key stakeholders’ opinions on feasibility, accessibility and sustainability of the proposed strategies. Their responses would be analysed and then used to fine tune the accepted strategies in preparation for implementation. The validation process is summarised on Fig. 4.

## Discussion

There have been no known studies found in literature that explores the possibility and developed strategies of integrating IHSs and MHSs so as to promote safe sexual practices in adolescents. Furthermore, no known studies were found that have explored the role that is played by IHS and MHS in shaping adolescent sexual experiences. Most programmes on sexual health have ignored the role of IHSs and MHSs in influencing safe sexual practices leading to them failing to attain desired goals. A lot of emphasis have been targeted at minimising the spread of Sexually Transmitted Infections (STIs) through advocating for utilisation MHSs rather than focussing on an integrating systems that are meant to manage Adolescent Sexual Health (ASH) related issues. This study might provide a window of opportunity in identifying IK, exploring IHS and MHS packages and how they influence sexuality in adolescents. If strategies are implemented they could improve ASH outcomes. This study is expected to generate at least 7 publications.

## Data Availability

Not applicable.

## Abbreviations

AIDS: Acquired Immunodeficiency Syndrome
BOEM: Build, Overcome, Explore and Minimise
FGDs: Focus Group Discussions
HIV: Human Immunodeficiency Virus
IHSs: Indigenous Health Systems
IK: Indigenous Knowledge
IKSs: Indigenous Health Systems
MHSs: Modern Health Systems
PHC: Primary Health Care
STIs: Sexually Transmitted Infections
SWOT: Strength, Weaknesses, Opportunities and Threats
WHO: World Health Organization

## References

[1] Macherera M, Chimbari MJ, Mukaratirwa S. Indigenous environmental indicators for malaria: a district study in Zimbabwe. Acta Trop. 2016. 10.1016/j.actatropica.2016.08.021 PMC5620432 27586040

[2] Aggleton P, Campbell C (2000). Working with young people-towards an agenda for sexual health. Sex Relatsh Ther.

[3] Edwards WM, Coleman E (2004). Defining sexual health: a descriptive overview. Arch Sex Behav.

[4] Health MO (2014). Sexual and reproductive Health needs of adolescents in Zimbabwe. International Perspectives on Sexual & Reproductive Health.

[5] Ismail S, Shajahan A, Sathyanarayana Rao TS, Wylie K (2015). Adolescent sex education in India: current perspectives. Indian J Psychiatry.

[6] Durie M (2005). Indigenous knowledge within a global knowledge system. Higher Education Policy.

[7] Marteleto L, Lam D, Ranchhod V (2008). Sexual behavior, pregnancy, and schooling among young people in urban South Africa. Stud Fam Plan.

[8] Kirby D (2002). The impact of schools and school programs upon adolescent sexual behavior. J Sex Res.

[9] Lam David, Marteleto Letícia J., Ranchhod Vimal (2013). The Influence of Older Classmates on Adolescent Sexual Behavior in Cape Town, South Africa. Studies in Family Planning.

[10] Lebese RT, Davhana-Maselesele M, Obi CL (2010). Sexual health dialogue between parents and teenagers: an imperative in the HIV/AIDS era. Curationis.

[11] Mulaudzi FM: Indigenous health beliefs, attitudes and practices among VhaVenda: A challenge to the promotion of HIV/AIDS prevention strategies, vol. 30; 2007.

[12] Maluleke TX: The puberty rites for girls (vukhomba) in the northern region of the Northern Province of South Africa : implications for women's health and health promotion. [Place of publication not identified]: [publisher not identified]; 2001.

[13] Mavundla TR, Netswera FG, Bottoman B, Toth F (2009). Rationalization of indigenous male circumcision as a sacred religious custom: health beliefs of Xhosa men in South Africa. Journal of transcultural nursing : official journal of the Transcultural Nursing Society.

[14] Care MHC (2016). Zimbabwe national adolescent fertility study.

[15] Moyo S (2013). Indigenous knowledge systems and attitudes towards male infertility in Mhondoro-Ngezi, Zimbabwe. Culture, Health & Sexuality.

[16] Agency ZNS (2011). Zimbabwe national Health profile.

[17] Del Fava E, Piccarreta R, Gregson S, Melegaro A (2016). Transition to parenthood and HIV infection in rural Zimbabwe. PLoS One.

[18] Magodoro IM, Esterhuizen TM, Chivese T (2016). A cross-sectional, facility based study of comorbid non-communicable diseases among adults living with HIV infection in Zimbabwe. BMC Res Notes.

[19] Mudonhi N, Nunu WN, Ndlovu B, Khumalo N, Dube O. Adolescents and parents’ perceptions of condom distribution in selected secondary schools in the high density suburbs of Bulawayo, Zimbabwe. Sexuality & Culture. 2019:1–19.

[20] Langhaug L, Cowan F, Nyamurera T (2003). Power on behalf of the Regai Dzive Shiri study group R: improving young people's access to reproductive health care in rural Zimbabwe. AIDS Care.

[21] Shumba K, Lubombo M (2017). Cultural competence: a framework for promoting voluntary medical male circumcision among VaRemba communities in Zimbabwe. African journal of AIDS research : AJAR.

[22] Moen T (2006). Reflections on the narrative research approach. Int J Qual Methods.

[23] Darroch JE. Research gaps in adolescent sexual and reproductive health. In: The Guttmacher Institute. 2016.

[24] Greer S, Wismar M, Figueras J (2016). Strengthening Health system governance : better policies, stronger performance.

[25] Hegamin-Younger C, Merrick J (2017). Caribbean adolescents : misuse and abuse of alcohol.

[26] Bassani C (2012). Adolescent behavior.

[27] Swartzendruber A, Zenilman JM (2010). A national strategy to improve sexual health. JAMA.

[28] Kinghorn G: A sexual health and HIV strategy for England: This ambitious strategy could, if properly resourced, greatly improve sexual health. In*.*: British Medical Journal Publishing Group; 2001.

[29] Denno DM, Hoopes AJ, Chandra-Mouli V (2015). Effective strategies to provide adolescent sexual and reproductive health services and to increase demand and community support. J Adolesc Health.

[30] Freedman L (2013). Strategy : a history.

[31] Mantula F, Saloojee H (2016). Child sexual abuse in Zimbabwe. Journal Of Child Sexual Abuse.

[32] Kmet LM, Cook LS, Lee RC: Standard quality assessment criteria for evaluating primary research papers from a variety of fields. 2004.

[33] Tofthagen R, Fagerstrøm LM: Rodgers' evolutionary concept analysis - a valid method for developing knowledge in nursing science R. Tofthagen, L.M. Fagerstrøm Presentation of Rodgers' evolutionary concept analysis. Scand J Caring Sci 2010, 24:21–31. 10.1111/j.1471-6712.2010.00845.x 21070310

[34] Shea BJ, Grimshaw JM, Wells GA, Boers M, Andersson N, Hamel C, Porter AC, Tugwell P, Moher D, Bouter LM (2007). Development of AMSTAR: a measurement tool to assess the methodological quality of systematic reviews. BMC Med Res Methodol.

[35] Twycross Alison (2004). Research design: qualitative, quantitative and mixed methods approachesResearch design: qualitative, quantitative and mixed methods approaches Creswell John W Sage 320 £29 0761924426 0761924426. Nurse Researcher.

[36] Magaya L (2010). Predictors of sexual risk behavior among Zimbabwean adolescents with and without disabilities: implications for HIV/AIDS prevention. Journal of International Special Needs Education.

[37] AIDS UN (2014). Zimbabwe developing subnational estimates of HIV prevalence and the number of people living with HIV.

[38] Sedgh G, Finer LB, Bankole A, Eilers MA, Singh S (2015). Adolescent pregnancy, birth, and abortion rates across countries: levels and recent trends. The Journal of adolescent health : official publication of the Society for Adolescent Medicine.

[39] ZIMPHIA (2016). Zimbabwe population -based HIV impact assessment.

[40] Hurst S, Arulogun OS (2015). Owolabi aO, Akinyemi R, Uvere E, Warth S, Ovbiagele B: pretesting qualitative data collection procedures to facilitate methodological adherence and team building in Nigeria. Int J Qual Methods.

[41] Maguire M, Delahunt B: Doing a thematic analysis: A practical, step-by-step guide for learning and teaching scholars. AISHE-J: The All Ireland Journal of Teaching and Learning in Higher Education 2017, 9(3).

[42] Almalki S (2016). Integrating quantitative and qualitative data in mixed methods research--challenges and benefits. Journal of Education and Learning.

[43] Creswell JW: Qualitative Inquiry and Research Design: Choosing among Five Approaches [with CD-ROM]. Second Edition: SAGE Publications (CA); 2006.

[44] Shento AK (2004). Strategies for ensuring trustworthiness in qualitative research projects. Educ Inf.

[45] Cleland J: Illustrative questionnaire for interview-surveys with young people, in: Cleland j, Ingham R and Stone N , eds, Asking young people About Sexual and Reproductive Behaviors, Illustrative Core instruments, Geneva: World Health Organisation. 2001.

[46] Hande S (2014). Strengths weaknesses opportunities and threats of blended learning: students' perceptions. Annals of medical and health sciences research.

[47] Van Durme T, Macq J, Anthierens S, Symons L, Schmitz O, Paulus D, Van den Heede K, Remmen R (2014). Stakeholders' perception on the organization of chronic care: a SWOT analysis to draft avenues for health care reforms. BMC Health Serv Res.

[48] Foundation WKK (2004). Using logic models to bring together planning, evaluation, and action: logic model development guide.

[49] Izaryk K, Skarakis-Doyle E (2017). Using the Delphi technique to explore complex concepts in speech-language pathology: an illustrative example from Children's social communication. American Journal Of Speech-Language Pathology.

[50] Njuangang S, Liyanage C, Akintoye A (2017). Application of the Delphi technique in healthcare maintenance. International Journal Of Health Care Quality Assurance.

